# Profile: David O'Flynn

**DOI:** 10.1192/bjb.2018.26

**Published:** 2018-08

**Authors:** Julia Bland

**Julia Bland meets Dr David O'Flynn**, the insider of ‘outsider’ art, Chair of The Adamson Collection Trust and Consultant Rehabilitation Psychiatrist in Lambeth.


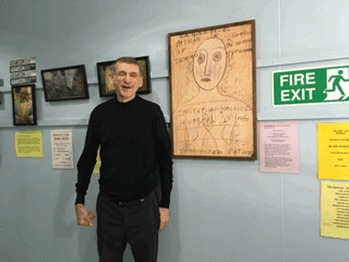


Dr O'Flynn contains within himself some of the contradictions of ‘outsider’ art. On first meeting, I unthinkingly proffered my right hand to shake his right hand, and then kicked myself as he offered his left hand: he has a right-sided hemiplegia. I don't think he was remotely bothered by the momentary awkwardness. From the beginning he exudes a joyous, almost boyish, enthusiasm; both for the works of The Adamson Collection and for his work on the Tony Hillis Unit, a 15-bed locked ward in Lambeth Hospital for men with chronic psychosis, behavioural problems and addictions. His sparky nature seems paradoxically linked to a very unfunny situation. Living with a terminal illness for much of his 30s, he has developed a sense of proportion about what really matters: ‘I shouldn't really be here ….. it's all extra’.

He enthuses about his work with intractable, sometimes violent, patients with psychosis, many of which have substance misuse problems in the mix. The atmosphere on the ward is calm and the patients are friendly, albeit with intense psychopharmacological treatment. They have an inside gym, an outside area and art sessions on the ward. ‘It's really a therapeutic community, exactly what I always wanted.’ The mean length of stay is 15 months. He remembers learning a slow, thoughtful approach to crisis on the ward from a consultant when he was a trainee. There had been a fight between five patients and ‘he taught me to go slowly, considering each patient, one by one.’

How did Dr O'Flynn get involved with The Adamson Collection?

He continued working in the emerging field of rehabilitation psychiatry, in spite of his poor health, and went to a psychiatric conference in Delphi where – by chance – he met a trustee of The Adamson Collection. O'Flynn was subsequently co-opted onto the committee that was planning Edward Adamson's legacy. Through this work, he became a personal friend of Adamson's surviving partner, John Timlin.

What is The Adamson Collection?^1^

Edward Adamson (1911–1996) was an artist who was employed at Netherne Hospital to run an art facility for the patients from 1946 until his retirement in 1981. Before he started, some psychiatrists had set up the art studio as a kind of experimental laboratory, for example to compare a patient's work pre- and post-lobotomy.

Adamson had a very different approach. He created a calm place, with classical music in the background, where patients could produce whatever they liked and there was no interpretation. He believed that making illustrations of any kind had a healing effect. As Anthony Stephens the Jungian analyst wrote: ‘Adamson enabled them to formulate the meaning of their predicament; and by mobilising the creative resources latent within their own personalities, he assisted them to heal themselves.’

He even managed to arrange separate spaces for patients who couldn't tolerate the studio with other people. He also enacted respect for each patient by providing a chair, an easel and a small table for paints and brushes so that each person had a distinct individual space within the room. This was in contrast to the uniformity of the institutional regime: beds lined up in a ward with no privacy.

Adamson was a trained artist and not a therapist; although he was influenced by Jungian ideas, regularly visiting the Jungian art therapy community Withymead in Oxfordshire. He had been a conscientious objector during the war and trained as a chiropodist. He established the British Association of Art Therapists in 1964, as well as the first art therapy training programme in 1969.

Particularly remarkable artists in The Adamson Collection (see below) include J. J. Beegan, who poignantly drew on lavatory paper with burnt matchsticks, and Mary Bishop, who expressed her utter despair and frustration with psychiatrists in paintings such as *Cri du Couer* and *Sadist in a White Coat*. Other notable workers in Adamson's studio include William Kurelek, a Canadian of Ukrainian origin who went on to be a successful, recognised artist later in his life. Rosanda Polonska spent 35 years in Netherne, producing drawings, poetry and sculpture, including the *Stations of the Cross* which were put up in the Netherne Hospital chapel. In 1982 she left hospital to live with her sister in Paris. Gwyneth Rowlands was completely original, making painted sculptures out of flints and pebbles she found. Some of these are permanently on display in Lambeth Hospital. Other Rowlands flints, pottery and other sculptural works are kept in The Adamson Collection Trust at the Bethlem Royal Hospital, and another 6 are in the Reading Room of the Wellcome Collection.

**Fig. 1:**
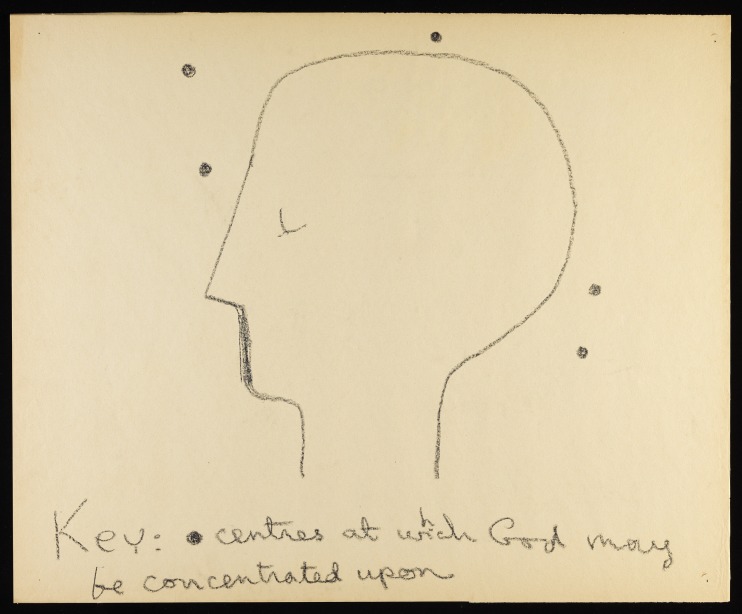
*Key Centres* by Martin Birch, date currently unknown. Pencil on paper. Courtesy of the Adamson Collection/Wellcome Library.

The thousands of works produced at Netherne underwent complicated selection processes. Adamson himself organised a group exhibition as early as 1947; and in 1956, 500 works were chosen by Adamson for the gallery he had established at Netherne. After he retired this became a physiotherapy room.

In 1981 Adamson and Rudolph Freudenberg selected several thousand works for storage and display at the entomologist Miriam Rothschild's Ashton Wold estate in Northamptonshire. When they later returned to Netherne, the rest of the works had disappeared.

In 1997 the whole collection was moved to Lambeth Hospital, which didn't have an adequate storage facility, and pieces were stored on open shelves and even in a disused shower cubicle!

**Fig. 2:**
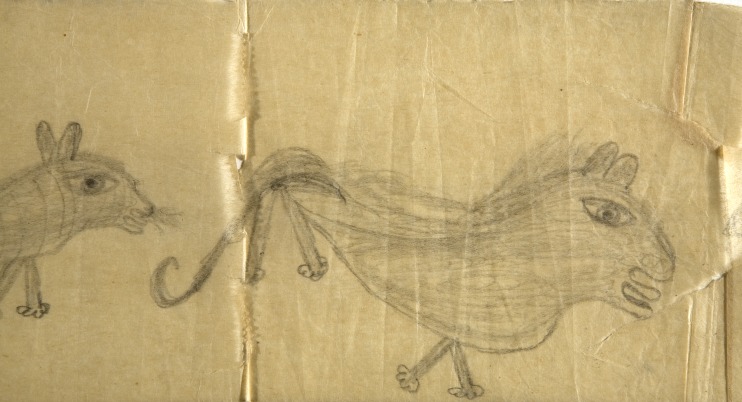
*Graffiti on Lavatory Paper 2: 3 Lions* (detail) by J. J. Beegan, c. 1946. Match char on Izal medicated toilet paper. Courtesy of the Adamson Collection/Wellcome Library.

Finally, the works on paper were accepted into the Wellcome Library in 2013, where they are now stored and curated.

In what ways does David O'Flynn think art therapies help? Is it an expression of distress and frustration without violence, respect for the production process or do the boundaries of the therapeutic group make it a safe place? Is it a validation of experience? Is it that the act of concentrating on producing an external concrete object, e.g. a painting, relieves the mind of tormenting internal preoccupations, allows distancing and an objectification of thoughts and feelings and thus provides a positive relief? Could engagement with the medium in a quiet place, with a soothing but non-judgemental parental presence, perhaps allow some reworking or reawakening of childhood playfulness?

In 1996 the Hayward Gallery exhibited ‘psychotic art’ from the German Prinzhorn Collection; the art consisted of poignant pieces by long-term institutionalised patients.^2^ Like The Adamson Collection, there are recurrent themes of fear, disintegration, fragmentation, feeling observed (multiple eyes), loss of control/being controlled (e.g. rays, pursuing objects, etc.), magical creatures and variable and multiple perspectives. The artworks feel like attempts at mastery of internal chaos.

**Fig. 3:**
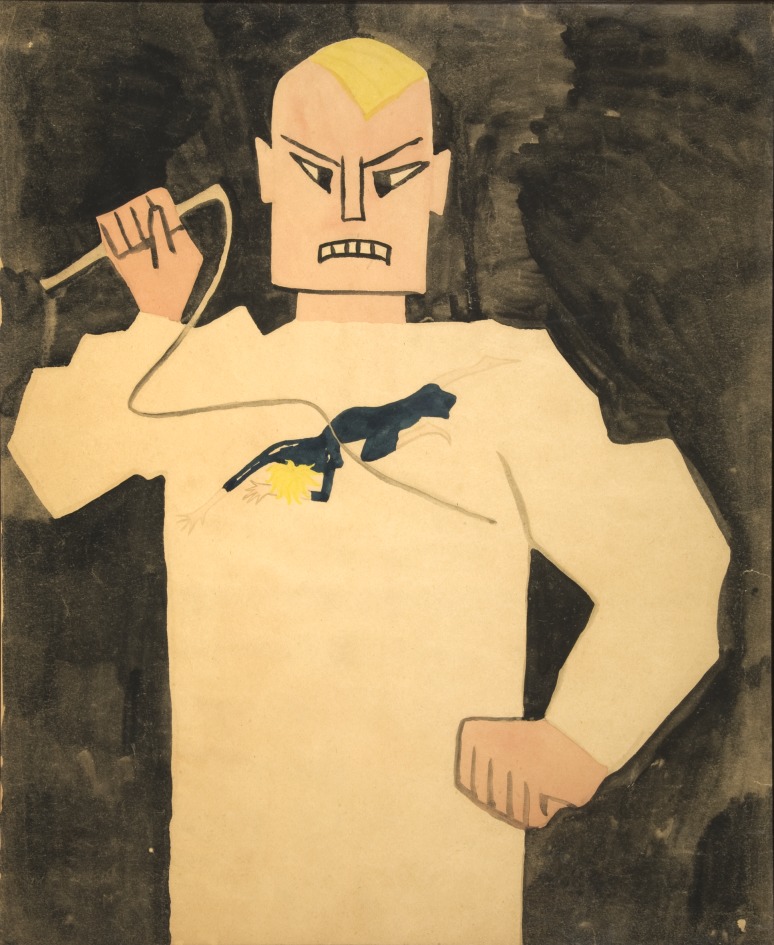
*The Sadist in the White Coat* by Mary Bishop, date currently unknown, probably late 1950s. Poster paint on paper. Courtesy of the Adamson Collection/Wellcome Library.

The past decade has brought a newer phenomenon of ‘community arts’: public arts associated with well-being that include drama as well as painting. This phenomenon seems to be targeted at a different patient group, i.e. people that are managing in the community, rather than the long-stay asylum population that Adamson was catering for.

David O'Flynn seems like an ideal fit for The Adamson Collection: interested in art, he used to escape the claustrophobic suburb as a child to look at pictures. When he was at school at Westminster, he often dropped into the Institute of Contemporary Arts, where he spotted his first punk. He lives with his husband in a not-yet-gentrified part of West London in a house full of reggae. He describes the area as ‘a real mix …  there are old ladies who still go to the shops in slippers and a dressing gown …’

He was brought up in respectable Blackheath, the son of a dedicated surgeon at Guy's Hospital. His brother is also a psychiatrist and is married with children. The young David was sent to Dulwich Preparatory School and then went on to Westminster from 1974–1978, an institution he appreciates for giving him ‘confidence without arrogance’. And for a final irony, long before he was aware of Adamson, he had a Jungian analysis with someone who was also an art therapist.

**Fig. 4 and 5:**
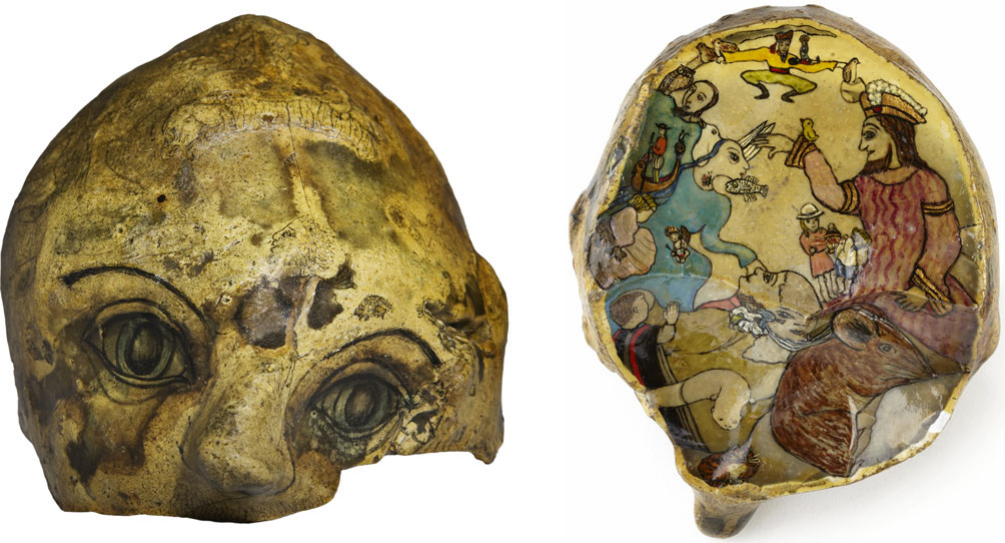
*Self-portrait: Skull Head* by Gwyneth Rowlands, date currently unknown. Vault and underside. Indian ink, watercolour and varnish on flint. Courtesy of the Adamson Collection Trust.

*Abandoned Goods* is a film that has been made about The Adamson Collection. It powerfully illustrates how the meaning of the works is radically altered by their location. Probably the most extreme example of this is how the work of J. J. Beegan, on lavatory paper, came to be the centre of an exhibition in Paris while on loan from the Wellcome Collection.

Within the film there is a marvellous vignette of a conversation between two (male) psychiatrists. First psychiatrist: ‘We haven't got a clue why they get ill, why they get better or what causes them to relapse.’ Second psychiatrist: ‘So we don't know anything about ourselves?’ First psychiatrist: ‘Yes that's probably true.’

I think that the works of The Adamson Collection challenge us as contemporary psychiatrists. When Adamson was working at Netherne, many patients were there for 30 years or more. They were subjected to physical restraint, overcrowding and neglect in many cases. Adamson's work was about respecting the individual as the author of the meaning of their experience.

Modern psychiatrists try to respect each individual but we have created the organisation of mental healthcare in such a fragmented fashion: the patient moves from team to team and sees different mental health professionals each time. We risk losing sight of the importance of the long-term, one-to-one relationship where patients feel respected rather than pushed from pillar to post.

Interestingly Dr O'Flynn's approach in the Tony Hillis Unit does allow time for each patient to be treated as an individual. So who better to be Chair of The Adamson Collection?

## About the author

**Julia Bland** is an honorary consultant psychiatrist for South London and Maudsley National Health Service Foundation Trust and a consultant medical psychotherapist at DocHealth, British Medical Association, UK. Email: julia.bland@hotmail.co.uk

## Further reading

http://www.adamsoncollectiontrust.org/resources/.
